# Food for Thoughts for Prospective Biomanufacturing

**DOI:** 10.1111/1751-7915.70088

**Published:** 2025-01-13

**Authors:** Ralf Takors

**Affiliations:** ^1^ Institute of Biochemical Engineering/Institut für Bioverfahrenstechnik University of Stuttgart Stuttgart Germany

## Abstract

While rising greenhouse gases cause climate change, global economies ask for resilient solutions for the business of the future. Biomanufacturing may well serve as a pillar of a circular economy with minimised environmental impact. Therefore, innovations of the lab need to successfully bridge the imminent ‘death‐valley of innovation’ for making commercial production happen. This editorial aims to prepare the ground for prospective developments so as to the seed of novel ideas will prosper.

## 1 The Need for Change

We are witnessing changes of climate and geopolitical structures. The well‐known Keeling curve (https://keelingcurve.ucsd.edu/), indicating the atmospheric CO_2_ content at Mauna Loa observatory, shows 424 ppm of the greenhouse gas which is about 50% more than during the pre‐industrialization era. At the same time, geopolitical changes endanger the supply chain of anyway limited fossil resources that still are the foundation of current chemical industry. Today, sustainability and resilience are important topics on the agenda of leading businesses as outlined by the World Business Council for Sustainable Development (https://www.wbcsd.org/).

Hence, the fossil based chemical industry needs to undergo a transition towards a sustainable economy. An important part of the solution is biomanufacturing that intrinsically builds on sustainable resources and on the valorization of side streams and waste. This is outlined by a Communication of the European Commission stating that ‘…Biotechnology and biomanufacturing are key for the competitiveness and the modernisation of our economy…’ (COM 2024). Likewise, biomanufacturing is strongly supported in the USA (White House Fact Sheet 2022) and in other regions around the globe.

While the need for transitioning the current industry to a sustainable economy is accepted the realisation is challenging. Regarding chemical industry any new biomanufacturing approach has to compete with existing infrastructure that was optimised steadily during the last 100 years. Furthermore, novel production approaches shall not assume that fossil based industry will diminish from 1 day to the other. Instead, oil and gas based chemical production is likely to remain for a long transitional period in those region with secured access delivering to local and global markets. As a consequence, biomanufacturing can only succeed if economic performance at least equals with the fossil products. Hoping for consumer concessions for higher prices of ‘green’ products may only work for product segments other than commodities and fine chemicals.

## 2 The Economic Production Scale

Shifting oil and gas based production of commodities and fine chemicals to sustainable biomanufacturing certainly offers a stronger leverage on climate protection than focusing on products with relatively low volumes. However, the latter may be more attractive for newcomers as relatively high profit margins are expected that even tolerate not fully optimised biomanufacturing approaches. In other words, establishing sustainable biomanufacturing processes for commodities and fine chemicals intrinsically demands for fully optimised production operating at theoretical limits in commercial scale. Any loss of production performance while transferring the process idea from the lab to industrial scale ultimately endangers the competitiveness of the industrial biomanufacturing approach. This is why successful scale‐up is particularly important for those products.

Experience of more than one century of chemical production taught us the advantage of economy‐of‐scale. The principle, which is also applicable for biomanufacturing, states that investment costs for large‐scale infrastructure do not rise proportionally with volume but follow the exponential increase with factor 0.6 instead. In other words, technical equipment 1000 fold larger than the reference size only costs about 63 fold more than the reference. Related savings not only reduce capital expenditures of investment (CAPEX) but also manufacturing costs via lowered depreciation impacts. Consequently, current biomanufacturing studies for commodities and fine chemicals are well advised to focus on large‐scale applications targeting 5–50 m^3^ for fine chemicals and > > 100 m^3^ for commodities.

In parallel, a novel movement gains momentum: the decentralised production exploiting the availability of local resources (e.g. ‘BioMADE’ concept, Attal‐Juncqua et al. 2024). Transportation costs are reduced and potentially fragile international supply chains are replaced by local substrate availabilities. To improve flexibility small scale production entities should ideally operate fully automated not requiring expert knowledge. Apparently, the successful implementation of such biomanufacturing approaches requires additional skills other than for centralised large‐scale production sites. The future will show how competitive likewise approaches will be.

## 3 The Feedstock Impact

The decision for the right feedstock for biomanufacturing is crucial. So‐called first generation (1G) feedstocks use glucose, glucose‐fructose mixtures and sucrose which are typically produced from sugar beets, sugar cane, corn and cassava in Europe, South America, North America and Asia, respectively. Besides, glycerol typically generated from palm oil, plays a role. Noteworthy, also crude substrates are used, such as molasses, thick juice and crude glycerol. 1G substrates are dominating industrial biomanufacturing. Often, production sites are found in proximity to the plant cultivation and their corn mills mirroring that much more substrates than products need to be transported.

Lignocellulosic substrates represent feedstocks of the 2nd generation (2G). Nutrient compositions typically comprise aromatic components of the lignin fraction, pentoses (and also glucose) of the hemicellulose and glucose from the cellulose part. Obviously, microbial producers must be able to deal with such complex sources. Often faced problems are the required tolerance to inhibiting components such as ferulic acid and metabolic engineering measures to enable new‐to‐the‐host pentose catabolism. Not to forget is the challenging handling and processing of the lignocellulosic biomass which is much more elaborative than utilising 1G feedstocks. Separating the lignocellulosic fraction to produce profitable compounds remains one of the challenges in second‐generation (2G) technology. Besides, industrial users thinking of utilising 2G feedstocks often face the problem of non‐secured supply for large‐scale biomanufacturing. Given the intrinsically reduced energy content of the lignocellulosic biomass compared to the 1G feedstocks much more material must be accessed, transported, and processed which represents a formidable challenge. Furthermore, resulting 2G substrate feeds typically show lower digestible sugar contents than 1G feeds which reduces production titers and poses additional problems on downstream processing.

The family of 3G feedstocks basically focuses on the valorization of CO_2_ and CO_2_ containing syngas compositions (CO_2_ + CO + H_2_). Given the high oxidation state of C (+4) in CO_2_, further electrons are needed to enable carbon metabolism in microbes. Under photoautotrophic conditions microalgae benefit from light as energy source. In contrast, anaerobic acetogens either co‐utilise H_2_ or prefer CO in metabolism. Native products of the latter are short chain organic acids (e.g. acetate) and alcohols (e.g. ethanol). Interestingly, those may well serve as substrates for follow‐up biomanufacturing which deems to be a promising approach for establishing a circular economy with minimised CO_2_ footprint. Alternately, combinations of electrochemical reduction with heterogeneous catalysis may be applied to produce short chain alcohols (e.g. methanol) or formiate from CO_2_ with electricity.

**FIGURE 1:**
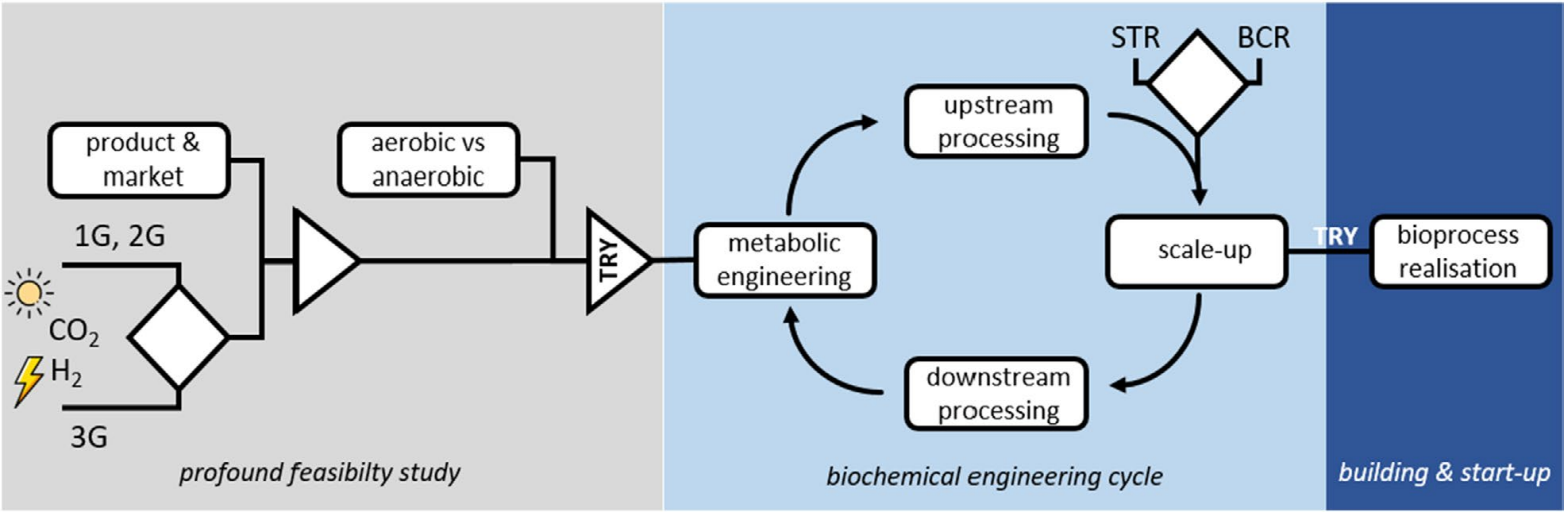
Realisation of novel biomanufacturing approaches. During the profound feasibility study fundamentals are evaluated regarding the product, the market, the feedstocks and the biomanufacturing concept. The biochemical engineering cycle integrates the key activities metabolic engineering, up‐ and downstream bioprocess development and also scale‐up. The latter should evaluate basic bioreactor concepts such as the stirred tank reactor (STR) versus bubble‐column type bioreactors (BCR). Only if benchmarks for critical TRY values (titers, rates, yields) are achieved project realisation continues.

The complexity of today's feedstock portfolio and its impact on biofuel production is outlined in Pfleger and Takors (2023). Hence, any novel biomanufacturing approach shall carefully decide on the best suitable feedstock not only because the decision heavily impacts the host selection and bioprocess development but also because feedstock availability is a matter of local constraints (see Figure 1).

## 4 Begin With the End in Mind

As simple as this statement (American book writer Stephen Covey) is as important it is also for the development of novel biomanufacturing processes (Noorman and Heijnen 2017; Straathof et al. 2019). Fundamental bioprocess decisions need to be made already at the beginning that are decisive for the weal and woe of the progress (see Figure 1). For instance, scientists should carefully consider whether aerobic or anaerobic conditions are preferred. With respect to the first, aerobic metabolism offers much more ATP availability for cellular product formation than anaerobic conditions. Regarding glucose catabolism, glycolysis plus respiration may provide about 16 ATP (For the sake of simplicity NADH is considered as the only reduction factor that is respired with the P/O ratio of 1.2, a rather conservative but realistic estimate that is far less than theoretical optima of 2 or even 3) compared to 2 ATP from anaerobic metabolism. Obviously, the microbial production of ATP‐intensive products benefits from respiration allowing higher biomass specific productivities in aerobic than in anaerobic conditions. Additionally, anaerobic production requires that electron balances are fully closed even without the drain of electrons to non‐desired by‐product formation. The disadvantage of low biomass specific product formation rates could be compensated by likewise increased biomass which either leads to increased biomass concentrations or larger tank volumes. Both are key factors for bioprocess design.

However, the decision for aerobic bioprocesses also challenges bioreactor design. Sufficiently high oxygen transfer rates (OTRs) typically larger than 120 mmol O_2_ L^−1^ h^−1^ need to be ensured even for high filling volumes. Furthermore, oxidation of NADH in the respiratory chain provides 220 kJ mol^−1^ free Gibbs energy (At standard conditions) that is released as heat in large quantities. Hence, proper cooling capacities need to be installed that get more and more challenging with rising bioreactor volumes.

Today, the dominating reactor setting is the stirred tank reactor. The essential tasks of mixing and mass transfer are split to stirring and aeration. Combinations of Rushton turbines, pitched blade, elephant ear impellers and others take the job to couple the mechanical energy into the liquid for mixing and the dispersion of bubbles. Power input via compressed air for aeration is less than for stirring. On the other hand, non‐mechanically agitated bioreactors such as bubble columns perform without any rotating device. Mixing and mass transfer are both realised via aeration and rising bubbles. Interestingly, bubble column type bioreactors (BCRs) can achieve similar mass transfer qualities than stirred tanks (i.e., *k*_*L*_ *a* coefficients) but require less power input. Additionally, considering the relatively simple design of BCRs, they offer operational and financial advantages (Humbird, Davis, and McMillan 2017) that are not yet reflected in the current status quo of the biotech industry. Though, the production of biomass, citric acid, 1,3 propane diol (Bisgaard et al. 2022) and formerly L‐phenylalanine (Mast, Ghaderi, and Takors 2024) convincingly showcase the large scale attractiveness, not to forget the successful application of BCRs in gas fermentation (Puiman et al. 2022).

## 5 Scaling Up

Novel ideas for biomanufacturing should not stay in the lab. Robust scaling‐up technologies should pave their way to make the bioprocesses happen in large scale. But, the majority of approaches still fails to bridge the so‐called ‘death‐valley of innovation’, unfortunately. Key performance indicators such as titre, rates and yields, often abbreviated as TRY values, do not fulfil economic expectations. Researchers shall be aware that high product titers are the prerequisite for eased downstream processing, rates are the synonym for high productivities, and yields mainly focus on high substrate‐to‐product conversion, a major impact factor to ensure highest carbon economy while preventing non‐wanted by‐product formation. Not to forget is the product quality that should fulfil the market expectations.

Successful bioprocess engineering should always aim to translate microbial production kinetics in bioprocess modes that aim for highest TRY levels. Ideally, maximum substrate‐to‐product conversion should coincide with high titers and productivities. Intrinsically, continuous operation holds great promise for the latter. But often, low biomass specific productivities impede this goal. In such cases, high volumetric productivity may still be achieved thanks to biomass retention (Castillo‐Saldarriaga et al. 2024).

The maturity of biomanufacturing is mirrored by the achievement of TRY thresholds that are individual for each product and its prospective market. Successful scaling‐up simply requires to accomplish the same TRY benchmarks also in commercial scale. Comprehensive reviews outline that physical, chemical, and biological factors differ between small and large scale finally creating stress conditions that may hamper cellular performance in industrial applications (Enfors et al. 2001; Lara et al. 2006; Garcia‐Ochoa and Gomez 2009; Takors 2012; Nadal‐Rey et al. 2021).

To mimic large‐scale mixing heterogeneities so‐called ‘scale‐down’ simulators (Neubauer and Junne 2010) may be well applied. Ongoing research for downscaling (Blöbaum, Haringa, and Grünberger 2023; Gaugler et al. 2024) makes them even applicable in early stage of strain and bioprocess development to select for robust hosts. Besides, the concept of ‘lifelines’ successfully aims to qualify and quantify the impact of changing microenvironmental conditions on the performance of producing cells (Haringa et al. 2016). Meanwhile, it is integral part of modern strain, bioreactor and bioprocess design (Delvigne et al. 2017; Wang et al. 2020).

Finally, researchers should be aware that cellular responses on large‐scale mixing heterogeneities are very individual. Regarding *Escherichia coli* the straightforward deletion of genes that showed non‐necessary on‐shifting under simulated large‐scale conditions lead to the robust host *E. coli* RM214 with superior production performance (Ziegler et al. 2021; Cordell et al. 2024). Other strains such as *Pseudomonas putida* KT2440 apparently offer intrinsic scale‐up advantages that may be exploited in future applications (Ankenbauer et al. 2020).

### 5.1 Last—but Not Least

Biomanufacturing shall not focus on upstream alone. Instead, research for downstream processing should be an integral part of the strain and process engineering already from the beginning (see Figure 1). Proper approaches for cell/liquid separation, extraction, chromatography, drying etc. should be tested whenever milestones are receivable, at least. Downstream processing provides essential feedback of manageable matrix compositions that impacts product titers, by‐products levels and the required purity of substrates. Hence, efforts for downstream processing should intensify with the maturity of strain and upstream engineering. Researches should be aware that downstream processing costs may account for more than 50% of total manufacturing expenses.

*Summarising*, shortening fossil resources, the need to establish a resilient economy and—maybe most important—the imperative to prevent human‐made climate change are driving forces strengthening our efforts for establishing a circular economy with biomanufacturing as a pillar. The portfolio of most valuable tools for biochemical engineering is rich. Researchers are not only invited to keep on developing new ones but also to rigorously apply most promising technologies to develop commercial applications of the next generation.

## Author Contributions

The author Ralf Takors has conceptualised and written the manuscript.

## Conflicts of Interest

The author declares no conflicts of interest.

## Data Availability

Data sharing not applicable to this article as no datasets were generated or analysed during the current study.
